# Infant abuse diagnosis associated with abusive head trauma criteria: incidence increase due to overdiagnosis?

**DOI:** 10.1093/eurpub/cky062

**Published:** 2018-04-17

**Authors:** Ulf Högberg, Erik Lampa, Göran Högberg, Peter Aspelin, Fredrik Serenius, Ingemar Thiblin

**Affiliations:** 1 Department of Women’s and Children’s Health, Uppsala University, Uppsala, Sweden; 2 UCR-Uppsala Clinical Research Centre, Uppsala University, Uppsala, Sweden; 3 Child and Adolescent Psychiatric Unit, Department of Women’s and Children’s Health, Karolinska Institute, Stockholm, Sweden; 4 Department of Clinical Science, Intervention and Technology (CLINTEC), Karolinska Institute, Stockholm, Sweden; 5 Forensic Medicine, Department of Surgical Sciences, Uppsala University, Uppsala, Sweden

## Abstract

**Background:**

The hypothesis of this study is that the diagnosis of infant abuse is associated with criteria for shaken baby syndrome (SBS)/abusive head trauma (AHT), and that that changes in incidence of abuse diagnosis in infants may be due to increased awareness of SBS/AHT criteria.

**Methods:**

This was a population-based register study. Setting: Register study using the Swedish Patient Register, Medical Birth Register, and Cause of Death Register. The diagnosis of infant abuse was based on the International Classification of Diseases, 9th and 10th revision. Participants: All children born in Sweden during 1987–2014 with a follow-up until 1 year of age (*N* = 2 868 933). SBS/AHT criteria: subdural haemorrhage, cerebral contusion, skull fracture, convulsions, retinal haemorrhage, fractures rib and long bones. Outcomes: Incidence, rate ratios, aetiologic fractions and Probit regression analysis.

**Results:**

Diagnosis of infant abuse was strongly associated with SBS/AHT criteria, but not risk exposure as region, foreign-born mother, being born preterm, multiple birth and small for gestational age. The incidence of infant abuse has increased tenfold in Sweden since the 1990s and has doubled since 2008, from 12.0 per 100 000 infants during 1997–2007 to 26.5/100 000 during 2008–2014, with pronounced regional disparities.

**Conclusions:**

Diagnosis of infant abuse is related to SBS/AHT criteria. The increase in incidence coincides with increased medical preparedness to make a diagnosis of SBS/AHT. Hidden statistics and a real increase in abuse are less plausible. Whether the increase is due to overdiagnosis cannot be answered with certainty, but the possibility raises ethical and medico-legal concerns.

## Introduction

In 1962 battered child syndrome was first described in a clinical study.^1^ In 1972 it was proposed that subdural haematomas could be caused by whiplash shaking^2^ and in 1974 the term ‘shaken baby syndrome (SBS)’ was coined to describe a condition inflicted by violent shaking and identified by the triad retinal haemorrhage, subdural haemorrhage and encephalopathy.^3^ The diagnosis of abusive head trauma (AHT), departing from the three diagnostic criteria for SBS, now has a broader categorization that may also include apnoea, seizures, fractures of the skull, metaphyses and shaft of long bones and ribs, and inability of parents/carers to provide an explanation for accidental trauma, the former findings and symptoms being considered highly specific for non-accidental trauma.^4–7^

Shaken baby syndrome/AHT is not a diagnosis classified in the International Classification of Diseases (ICD-9/ICD-10); however, physical abuse and battered baby or child syndrome is defined as maltreatment syndrome. In an intercountry epidemiological study, Gilbert et al. described the maltreatment syndrome/assault and included in their classification the diagnoses of intracranial injury and long bone fractures.^8^

The incidence of maltreatment syndrome has shown a variation by country,^8^ from 11.5 per 100 000 infants in Sweden (1987–2009), to 34.6 in England (1997–2008), to 118.9 in Western Australia (1980–2005), with only England showing a declining trend.^8^ The incidence of non-fatal AHT among infants, based on the case definition of the US Center of Disease Control and Prevention (CDC), was 32.3 per 100 000,^9^ while a Canadian study on infant AHT found an incidence of 13.0–15.5 for 2002–2007.^10^ In Scotland in 1998–1999, the incidence of shaken impact syndrome among infants was 24.6 per 100 000.^11^

Shaken baby syndrome has, since 1997,^12^ been questioned as a diagnostic entity, and emerging imaging technology demands further differential diagnostic considerations.^13–18^ Furthermore, the precision of the SBS/AHT diagnosis is lost when excluding the diagnostic criterion of incompatibility between the carer’s report of history and the investigating doctor’s interpretation of the findings.^19^ In 2016, a systematic literature review by the Swedish Agency for Health Technology Assessment and Assessment of Social Service (SBU) concluded that there is limited scientific evidence that the triad can be explained by isolated shaking, and that there is insufficient evidence to assess the diagnostic accuracy of the triad to identify SBS/AHT.^20^^,^^21^

In Sweden, battered child syndrome was introduced as a diagnostic criterion during the 1960s,^22^ and the triad of SBS was described in the *Swedish Medical Journal* in 1994.^23^ In 2008, Stockholm County Council published clinical guidelines for the investigation of SBS according to the AHT criteria and the document has been adapted for use in other parts of Sweden.^24^ In 2009, the Swedish Paediatric Society established a child abuse task force to foster awareness of and training in child abuse recognition and reporting, and implementation of the guidelines for investigation and diagnosis of AHT. Subsequently, regional hospital-based child protection centres were established.

This study examines the incidence of diagnosis of infant abuse over three decades in Sweden. The hypothesis of our study is that diagnosis of infant abuse is based on SBS/AHT criteria, and that changes in incidence of diagnosis of infant abuse may be due to increased awareness of SBS/AHT criteria.

## Methods

This is a population-based register study of children born in Sweden between 1987 and 2014, with a follow-up until 1 year of age.

The number of children born during the period 1987–2014 (*N* = 2 984 813) was retrieved from Statistics Sweden. The research database was linked to the Swedish Patient Register, the Swedish Medical Birth Register and the Swedish Cause of Death Register, with diagnoses and conditions classified using ICD-9 (1987–1996) and ICD-10 (1997–2015) codes. Infant abuse was defined according to the Swedish version of the ICD-9 and ICD-10 (table 1). For the years 1997–2014, ICD-10 codes for other possible differential diagnoses (table 1), symptoms and conditions which might be considered when investigating infant abuse, were searched for.^6^^,^^7^^,^^13^ In all, 182 974 infants with diagnostic codes were found, for each a control was selected, born in the same year and without any diagnosis in the Patient Register (*N* = 731 901). In total, the sample consisted of 914 875 infants, 49% of all infants born during 1997–2014. For this study, we selected as criteria for SBS/AHT to be associated with abuse diagnosis:subdural haemorrhage, cerebral contusion, skull fracture, convulsions, retinal haemorrhage, fractures of rib and long bones (table 1).^4^^,^^6–8^^,^^20^

**Table 1:** Definitions of infant abuse, and other diagnoses, according to the Swedish Patient Registry, National Board of Health and Welfare (ICD-9: 1987–1996; ICD-10: 1997–2014)

Diagnosis category		ICD code
Infant abuse diagnosis		
	Observation for suspected	Z03.8K (ICD-10); E967 (ICD-9)
	abuse	
	Battered baby syndrome	Y07.9 (ICD-10); 995F (ICD-9)
	Maltreatment syndrome	T74.1 (ICD-10); 995F (ICD-9)
	Neglect and adandonment	Y06
	Other maltreatment	Y07
Subdural haemorrhage		I62.0, S06.5 (ICD-10)
Skull fracture		S02.0, S02.1, S02.8, S02.09 S02.00, S02.9 (ICD-10)
Cerebral contusion		S06.0, S06.1 (ICD-10)
Convulsions		R56, R56.8, G40–41, R56.0 (ICD-10)
Retinal haemorrhage		H35.6 (ICD-10)
Rib fracture		S22.3, S22.4 (ICD-10)
Long bone fracture		S42.2, S42.3, S42, 4, S42.7, S42.8, S52, S72, S82, T10, T12 (ICD-10)

The baseline descriptive analysis was based on the incidence proportion of diagnosis of infant abuse per 100 000 by year (moving annual average). For thorough analysis of diagnosis, only years with ICD-10 coding were selected, 1997–2014. We based our analyses on incidence of diagnosis in the population and, for cases diagnosed with abuse, for the whole period divided by the two periods 1997–2007 and 2008–2014, before and after the increased awareness of SBS/AHT criteria was established in the Swedish setting. Diagnoses were selected by the incidence proportion per 100 000 [95% confidence interval (CI)], and the two periods were compared in terms of rate ratios (I_1_/I_0_) (95% CI) and aetiologic fractions of incidence (I_1_-I_0_/I_1_) (%). The following covariates were selected for further analysis because of a proposed association with the diagnosis of infant maltreatment: maternal country of birth; infant perinatal characteristics such as multiple birth, preterm birth (born at <37 weeks’ gestation), born Small-for-Gestational-Age (SGA) [<2.5 standard deviation (SD)]; diagnosis by health care region.

Probit regression analysis was performed in a Structural Equation Modelling (SEM) framework, in order to examine a conceptual model to explore the interplay of the variables of importance for the outcome ‘diagnosis of infant abuse’. The conceptual model is shown in figure 2. Implicit in the model is a latent diagnosis variable that is observed across the different diagnoses. The importance of the different observed diagnoses for the latent diagnoses variable was assessed by the factor loadings, with higher loading values implying greater importance.

Probit regression, like logistic regression, models the probability of an outcome variable being equal to one of two values and can be written as:

$$P\left(Y=1||X\right)=\Phi \left(X^{T}\beta \right),$$

where **X** is the data matrix and *β* is the vector of regression coefficients. Φ denotes the cumulative distribution function of the standard normal distribution. The coefficient of the latent diagnosis variable in the SEM Probit model should be interpreted as a change in z-score which allows calculation of the probability of an SD increase in the latent diagnosis variable. To investigate whether associations changed over time, the SEM model was divided into two groups defined by the time period and the coefficients compared.

The statistical software package IBM SPSS 25.0 (SPSS Inc., Chicago, IL, USA) and R version 3.3.1^25^ were used for data analyses. The SEM models were fitted using the lavaan add-on package for R.^26^

The study was approved by the Regional Ethical Committee in Uppsala (2014-11-19 No. 383).

## Results

**Figure 1:**
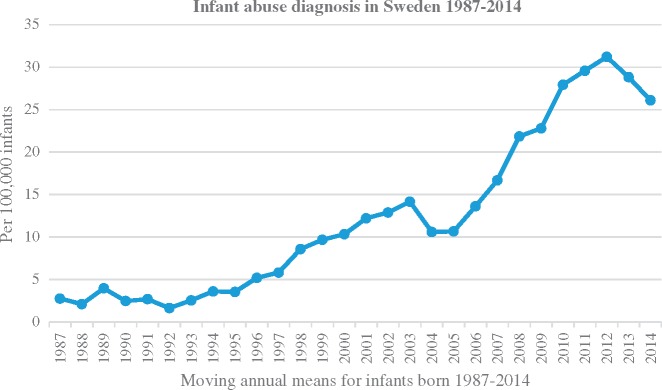
Infant abuse diagnosis in Sweden for children born in 1987–2014 per 100 000 infants (moving annual average)

Altogether 368 infants were diagnosed with maltreatment during the years 1987–2014; 12.3 per 100 000 infants. Seven of the children diagnosed with abuse died, i.e. 0.23 deaths per 100 000 infants and a case fatality rate of 1.9%*.* A tenfold increase in diagnosis of infant abuse was observed from the start to the end of the period. The increase started slowly from 1996, peaking during the years 2008–2014 (figure 1). There was a doubling of the incidence of abuse diagnosis during the years 2008–2014 compared with 1997–2007, from 12.0 (*n *= 127)–26.5 (*n *= 210) per 100 000 infants [odds ratio (OR) 2.21 (95% CI 1.78–2.76)].

Of the 337 cases of infant abuse during the years 1997–2014, 137 had either concomitant diagnoses of subdural haemorrhage, skull fracture, cerebral contusion, traumatic brain injury, convulsions, retinal haemorrhage, rib fracture, or fractures of the long bones. Out of the residual, 200 cases, 11 had injury to the head and 14 to the body, nine had injury of the eye or orbital fracture, five had a fractured clavicle, three had burns, three had diagnosis of failure to thrive; others had miscellaneous diagnoses such as infections, skin disease, nausea, obstipation and icterus. Altogether 125 had no other diagnosis besides abuse (table 1), whereof 60 had the diagnosis ‘observation for suspected abuse’ and 35 had a diagnosis of maltreatment syndrome.

**Figure 2:**
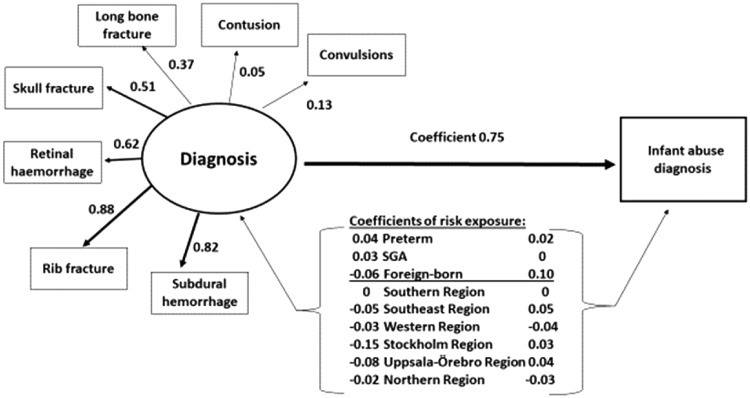
Conceptual model for diagnosis of infant abuse in Sweden in 1997–2014, performed in a Structural Equation Modelling (SEM) framework. Observed variables are given inside rectangles. The latent diagnosis variable is drawn as an ellipse. Arrows radiating out from the ellipse and pointing to the observed diagnoses represent factor loadings. Coefficients represented by arrows pointing to ‘Infant abuse diagnosis’ should be interpreted as the increase in z-score for the probability of observing the outcome. For the risk exposures, the coefficients represent the increase in z-score if the condition is present. For the latent diagnosis variable, the coefficient represents the increase in z-score for a standard deviation (SD) increase in the latent diagnosis variable. The coefficient for the Southern health region was set to zero as a reference for the other health regions. SGA = small for gestational age

The results of the Probit regression analysis are presented in figure 2 and Supplementary table S1. Four of the observed diagnoses: subdural haemorrhage, rib fracture, retinal haemorrhage and skull fracture, had substantial loadings on the latent diagnosis variable suggesting that these four diagnoses were the main drivers of the association with the probability for diagnosis of infant abuse. All diagnoses taken together were strongly associated with this probability [coefficient 0.752 (95% CI 0.709–0.795 (*P* < 0.01)]. If the baseline probability of diagnosis of infant abuse is around 0.01% with all other factors held at their reference values, an SD increase in the latent diagnosis variable would push that probability to Φ (−3.72 + 0.75) = 0.15%. Coefficients for diagnoses were increased for being born preterm and SGA. Coefficients for diagnosis of infant abuse were increased when being born preterm and having a foreign-born mother.

With the exception of convulsions, no statistically significant changes in population incidence of subdural haemorrhage, skull fracture, cerebral contusion, retinal haemorrhage, rib fracture or fractures of the long bones could be seen between the two periods 1997–2007 and 2008–2014 (table 2). However, cases diagnosed with infant abuse with those diagnoses had statistically significantly increased rate ratios, during 2008–2014, for subdural haemorrhage, skull fracture, retinal haemorrhage and long bone fractures; increased, though non-significant, rate ratios for cerebral contusion and convulsions; and aetiologic fractions ranging from 26.4% to 58.5% (table 2). Having an abuse diagnosis and being preterm born, Small-for-Gestational-Age or a twin was associated with greatly increased incidences during the latter period, with statistically significant rate ratio changes and aetiologic fractions ranging from 32.6% to 70% (table 2). Both infants of Swedish mothers and infants of foreign-born mothers had increased risk ratios in the latter period, especially infants of foreign-born mothers. The distribution of cases of infant maltreatment was uneven by region and period, with threefold geographical differences. No significant changes in the coefficients from the SEM were observed when comparing the models for the separate time periods.

**Table 2:** Diagnosis and background characteristics of infants or children with maltreatment syndrome in Sweden, 1997–2007 (*n* = 127) and 2008–2014 (*n* = 210),* per 100 000 children below 1 year of age (1997–2007 *n* = 1, 062, 084; 2008–2014 *n* = 793, 183)^‡^

Diagnosis and background Characteristics	Abuse diagnosis	1997–2007		2008–2014		2008–2014/1997–2007	
		*n*	Incidence (95% CI)	*n*	Incidence (95% CI)	RR (95% CI)	EFe (%)
Subdural haemorrhage	No	130	12.3 (10.4–14.5)	133	16.8 (14.2–19.7)		
	Yes	20	1.89 (1.22–2.79)	23	2.90 (1.94–4.20)	1.36 (1.07–1.73)	26.4
Skull fracture	No	876	82.5 (77.2–88.0)	612	77.1 (71.3–83.4)		
	Yes	12	1.13 (0.65–1.85)	21	2.65 (1.06–2.8)	2.34 (1.15–4.76)	57.3
Cerebral contusion	No	5357	504 (491–518)	3, 731	470 (455–486)		
	Yes	4	0.37 (0.15–0.83)	5	0.63 (0.28–1.29)	1.67 (0.45–6.23)	40.3
Convulsions	No	7225	680 (665–696)	5, 931	747 (729–767)		
	Yes	7	0.66 (0.33–1.23)	11	1.39 (0.78–2.32)	2.10 (0.82–5.43)	52.5
Retinal haemorrhage	No	70	6.59 (5.22–8.22)	49	6.18 (4.68–8.02)		
	Yes	10	0.94 (0.52–1.61)	18	2.27 (1.44–3.43)	2.41 (1.11–5.22)	58.5
Long bone fractures	No	1157	108 (103–115)	993	125 (117–133)		
	Yes	22	2.07 (1.37–3.02)	35	4.41 (3.18–5.99)	2.13 (1.25–3.63)	53.1
Rib fracture	No	26	2.44 (1.68–3.47)	20	2.52 (1.64–3.74)		
	Yes	12	1.13 (0.65–1.85)	20	2.01 (1.25–3.12)	2.32 (1.09–4.57)	55.2
Preterm-born (<37 wks)	Yes	18	27.3 (17.3–41.3)	27	57.2 (39.4–80.6)	2.09 (1.15–3.80)	52.2
Small for gestational age	Yes	7	29.6 (14.6–55.3)	8	43.9 (22.6–79.2)	1.48 (0.54–4.09)	32.6
Multiple birth	Yes	6	38.0 (17.9–74.0)	14	127 (76.2–202)	3.34 (1.28–8.68)	70.0
Mother Swedish-born	Yes	96	11.1 (9.13–13.5)	124	12.3 (12.4–18.5)	1.87 (1.43–2.44)	46.4
Mother foreign-born	Yes	31	15.6 (11.0–21.6	86	44.6 (35.9–54.2)	2.71 (1.80–4.09)	63.1
Southern Region	Yes	27	13.2 (9.07–18.6)	53	33.4 (25.6–43.0)	2.54 (1.60–4.03)	60.6
South East Region	Yes	22	20.4 (13.5–29.7)	19	24.2 (15.5–36.2)	1.59 (0.86–2.94)	37.1
Western Region	Yes	13	7.2 (4.24–11.6)	32	20.8 (14.4–29.1)	2.89 (1.49–5.57)	65.3
Uppsala-Örebro Region	Yes	36	17.3 (12.5–23.4)	32	24.4 (17.3–33.6)	1.41 (0.87–2.27)	29.2
Stockholm Region	Yes	16	6.00 (3.72–9.28)	72	34.9 (27.7–43.8)	5.81 (3.38–9.81)	82.9
Northern Region	Yes	12	12.8 (7.41–21.1)	5	10.8 (4.74–22.0)	0.21 (0.07–0.59)	–

Notes: Incidence proportion per 100 000 infants [95% confidence intervals (CIs)], rate ratio (RR) 2008–2014/1997–2007 and aetiologic fraction among the exposed (EFe), those born preterm or small for gestational age (SGA) and multiple births, for having an abuse diagnosis. *Source: Swedish National Board of Health and Welfare; ^‡^Source: Statistics Sweden and the National Board of Health and Welfare.

## Discussion

This study shows that diagnosis of infant abuse was strongly associated with SBS/AHT criteria. Over 27 years, the diagnosis of infant abuse in Sweden increased tenfold from the late 1980s to 2014, doubling from 12.0 per 100 000 infants during the period 1997–2007–26.5 during the period 2008–2014. Pronounced regional differences were observed. The doubling incidence was attributed to the SBS/AHT criteria, region, being preterm born or SGA and multiple births.

Our results support the interpretation that diagnosis of infant abuse is associated with SBS/AHT criteria. The high Swedish national incidence of infant abuse diagnosis is still lower, even during the latter period, than the infant maltreatment diagnosis reported from the USA,^27^ Canada^10^ and the UK,^8^ but is comparable with Scotland’s incidence of shaken impact syndrome.^11^

The increasing incidence in Sweden over time is intriguing. How is it that Sweden, previously having one of the lowest rates of infant maltreatment in Western societies,^8^ has manifested this increase? Is it because previous years, i.e. the 1980s and early 1990s, were characterized by underdiagnosis and hidden statistics, or is the increase due to overdiagnosis? Alternatively, has there been a real increase in infant abuse?

Whether there is an increase in true positive or in false positive cases, the Swedish doubling in cases coincides with the fact that there has been an increased awareness of SBS/AHT among Swedish paediatricians during the latter period of our study,^24^^,^^28^ and that doctors may therefore have become more likely to make an infant abuse diagnosis. A similar findingwas reported from a New Zealand hospital study where the incidence of diagnosis of AHT quadrupled from 1991 to 2010 after the establishment of a specialist child protection team; however, the possibility of overdiagnosis was not discussed.^29^ Unlike our national data for the years 2008–2014, national statistics from New Zealand on infant maltreatment-related injury admissions did not show an increase during the years 1995–2010^8^ contrary to the hospital study.^29^

One argument proposed for the hidden statistics hypothesis is a previously low identification rate due to a low proportion of Swedish paediatricians receiving training on child abuse,^24^ and that there are hidden cases of child abuse among infant deaths classified as ‘unknown cause of death’.^30^ However, this hypothesis was disproved in a recent study where all records of infants deceased in 1994–2013 were analyzed for diagnosis of AHT, and where, compared with international statistics on AHT death, a tenfold lower incidence is reported.^31^ The low case fatality rate in our study, 1.9%, does not correspond to the AHT fatality rate of 30% reported from hospital settings in the USA.^32^ Irrespective of the true aetiology of the lethal intracranial lesions, this disparity does not support hypotheses of either hidden statistics or a real increase, but supports the hypothesis of overdiagnosis.

There are several arguments supporting the overdiagnosis hypothesis. The regional variation suggests that the diagnosis of infant abuse is due to differences in applying the SBS/AHT criteria. In Stockholm, cases of infant abuse began to rise steeply, concomitant with the establishment of a child protection team and the introduction of a ‘mental vaccination’ against shaking programme in 2008–2010.^28^ Similar intervention programmes in the USA have reported decreased incidence of AHT.^33^ A possible interpretation of a contrary effect of the Swedish campaigns is that the increase in diagnosed cases of infant maltreatment did not indicate a real increase in incidence, but instead, a prevalence of overdiagnosis because of enhanced awareness and preparedness of SBS/AHT among health care professionals. Further, population surveys showed that Swedish parents reported shaking their children in 18% in 2006, while after an information campaign against shaking, parental reporting of shaking was reduced to almost nil by 2011.^34^

The diagnostic process of SBS/AHT has been, and still is, mainstream thinking in health care and considered a scientific fact,^7^ but this position has been critically challenged for the past two decades^13–16^ and the evidence has now been contested by the first independent systematic literature review ever completed regarding this diagnosis.^20^^,^^21^

Whether the increase in diagnosis of infant abuse is due to overdiagnosis, i.e. changes in diagnostic classification, this comprises ethical principles of beneficence, non-maleficence and justice. In the USA, the sum of infant homicide rates and accident mortality rates remained constant during the years 1980–2005, while the proportion of infant accident mortality rates to infant homicide rates decreased compared with the period 1940–1979. This may be explained by the changed diagnostic classification, since 1980, with more diagnosis of homicide cases and fewer cases of accidents being diagnosed.^35^ The possibility of overdiagnosis as a reason for the increase observed in the present study raises medico-ethical concerns relating to harm in health care, as non-evidence-based knowledge being practised and may affect families seeking health care for their infants.

How to interpret the strong association between perinatal risk exposure and infant abuse diagnosis in this study, especially in relation to the steep increase in abuse incidence during the latter period? One explanation could be the risk of metabolic bone disease in relation to being born preterm or SGA or being twin-born.^36–38^ Whether preterm and multiple births are more prone to having a birth-related subdural haemorrhage has not yet been reported. Foreign-born mothers had a higher incidence risk ratio, and a higher attributable risk, than Swedish-born mothers, and further displayed a higher risk coefficient for abuse diagnosis but not for AHT diagnosis compared with Swedish-born mothers. The reasons for this could not be ascertained in this study.

Differential diagnostic to abuse diagnosis of the SBS/AHT criteria applied in this study was not part of the study aim but the extent is displayed in table 2, and will be further analyzed in forthcoming studies.

### Strengths and limitations

A strength of the present study is that the health registers covered the whole country during the study period. More than 1/3 of cases of infant abuse during the years 1997–2014 had no additional code explaining the diagnosis of abuse. This is a major limitation in that we had no access to the individuals’ details and no checks had been made of clinical records, laboratory tests, or radiological or imaging reports. This hindered the possibility to establish to what extent diagnostic criteria, more non-symptomatic signs as retinal haemorrhage, thin subdural haemorrhage, classic metaphyseal lesions, or non-dislocated rib fractures, had been missed or how they had been interpreted in different ways. Neither we know whether there is underreporting of ICD-codes on abuse of children being subjected for intervention of Social Service. However, the direction of information and detection bias might lead to underestimation of the findings of this study. One further limitation is the change from ICD-9 to ICD-10, which may have affected incidence changes. However detailed analysis was solely restricted to ICD-10 codes*.* Although the conceptual Probit regression model fit was reasonable, with a comparative fit index of 0.86 and a root mean square error of approximation of 0.004, the model did not account for the information in the observed covariance matrix (*P* < 0.001).

## Conclusions

This study shows that the incidence of diagnosis of infant abuse is strongly associated with the diagnostic criteria of SBS/AHT; further, that the increase in incidence of diagnosis of abuse coincides with increased preparedness of doctors to make an SBS/AHT diagnosis. Hidden statistics or a real increase are a less plausible explanation. Whether the increase is due to overdiagnosis cannot be answered with certainty by this study, but the possibility that overdiagnosis of cases may be a reason for the increase does raise ethical concerns for medico-legal assessment in the context of child protection.

## Supplementary data

Supplementary data are available at *EURPUB* online.

## Supplementary Material

Supplementary Data
