# Supplementary material for: Infant abuse diagnosis associated with abusive head trauma criteria: incidence increase due to overdiagnosis?
Source: Eur J Public Health. 2018 Apr 17;28(4):641–6. doi: 10.1093/eurpub/cky062 (PMC6296307; doi:10.1093/eurpub/cky062)
Supplement: Supplementary Data [file cky062_supplementary_table.doc]

**Supplementary Table S1.** Probit regression analysis performed in a Structural Equation Modelling (SEM) framework for diagnosis of infant abuse in Sweden, 1997-2014. Latent diagnoses, all abusive head trauma (AHT) criteria, are explored for their loadings on the diagnosis of infant abuse. Z-scores show the probability of an increase in the latent diagnosis variable. Probit regression coefficients are estimates of the probability for the diagnosis of infant abuse for the group, and for those with risk exposure in terms of perinatal characteristics, by health region. SE = standard error; SGA = Small-for-Gestational-Age.

|  |  | **Estimate** | **SE** | **z-value** | **P(>**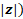 |
| --- | --- | --- | --- | --- | --- |
| **Latent diagnosis variables** |  |  |  |  |  |
|  | Retinal haemorrhage | 0.617 | 0.034 | 18.36 | <0.001 |
|  | Subdural haemorrhage | 0.815 | 0.02 | 41.49 | <0.001 |
|  | Rib fracture | 0.881 | 0.027 | 32.96 | <0.001 |
|  | Skull fracture | 0.512 | 0.016 | 32.34 | <0.001 |
|  | Long bone fracture | 0.368 | 0.018 | 20.95 | <0.001 |
|  | Cerebral contusion | 0.053 | 0.021 | 2.54 | 0.011 |
|  | Convulsions | 0.125 | 0.014 | 8.94 | <0.001 |
| **Regression coefficients for diagnoses** |  |  |  |  |  |
|  | Preterm | 0.037 | 0.005 | 7.75 | <0.001 |
|  | SGA | 0.032 | 0.005 | 6.89 | <0.001 |
|  | Mother foreign-born | -0.061 | 0.006 | -10.81 | <0.001 |
|  | Southern Region | 1 |  |  |  |
|  | South East Region | -0.049 | 0.006 | -8.57 | <0.001 |
|  | Western Region | -0.32 | 0.006 | -5.7 | <0.001 |
|  | Stockholm Region | -0.145 | 0.008 | -19.3 | <0.001 |
|  | Uppsala-Örebro Region | -0.082 | 0.006 | -12.89 | <0.001 |
|  | Northern Region | -0.024 | 0.005 | -4.53 | <0.001 |
| **Regression coefficients for infant abuse diagnosis** |  |  |  |  |  |
|  | Diagnoses for infant abuse diagnosis | 0.752 | 0.022 | 34.02 | <0.001 |
|  | Preterm | 0.01 | 0.011 | 1.9 | 0.057 |
|  | SGA | 0 | 0.01 | 0.012 | 0.991 |
|  | Mother foreign-born | 0.095 | 0.013 | 7.16 | <0.001 |
|  | Southern Region | 1 |  |  |  |
|  | South East Region | 0.051 | 0.014 | 3.59 | <0.001 |
|  | Western Region | -0.038 | 0.019 | -2.05 | 0.04 |
|  | Stockholm Region | 0.076 | 0.018 | 4.25 | <0.001 |
|  | Uppsala-Örebro Region | 0.04 | 0.017 | 2.36 | 0.018 |
|  | Northern Region | -0.0027 | 0.013 | -1.53 | 0.126 |
